# Epigenome wide association study of SNP–CpG interactions on changes in triglyceride levels after pharmaceutical intervention: a GAW20 analysis

**DOI:** 10.1186/s12919-018-0144-7

**Published:** 2018-09-17

**Authors:** Jenna Veenstra, Anya Kalsbeek, Karissa Koster, Nathan Ryder, Abbey Bos, Jordan Huisman, Lucas VanderBerg, Jason VanderWoude, Nathan L. Tintle

**Affiliations:** 1 Department of Biology, Dordt College, 498 4th Ave. NE, Sioux Center, IA 51250 USA; 2 Department of Mathematics and Statistics, Dordt College, 498 4th Ave. NE, Sioux Center, IA 51250 USA; 3 Department of Computer Science, Dordt College, 498 4th Ave. NE, Sioux Center, IA 51250 USA

## Abstract

In the search for an understanding of how genetic variation contributes to the heritability of common human disease, the potential role of epigenetic factors, such as methylation, is being explored with increasing frequency. Although standard analyses test for associations between methylation levels at individual cytosine-phosphate-guanine (CpG) sites and phenotypes of interest, some investigators have begun testing for methylation and how methylation may modulate the effects of genetic polymorphisms on phenotypes. In our analysis, we used both a genome-wide and candidate gene approach to investigate potential single-nucleotide polymorphism (SNP)–CpG interactions on changes in triglyceride levels. Although we were able to identify numerous loci of interest when using an exploratory significance threshold, we did not identify any significant interactions using a strict genome-wide significance threshold. We were also able to identify numerous loci using the candidate gene approach, in which we focused on 18 genes with prior evidence of association of triglyceride levels. In particular, we identified *GALNT2* loci as containing potential CpG sites that moderate the impact of genetic polymorphisms on triglyceride levels. Further work is needed to provide clear guidance on analytic strategies for testing SNP–CpG interactions, although leveraging prior biological understanding may be needed to improve statistical power in data sets with smaller sample sizes.

## Background

Methylation plays a major role in gene regulation through epigenetic modifications at specific cytosine-phosphate-guanine (CpG) residues within the regulatory regions of genes and, consequently, may influence the transcriptional activity [1]. In brief, methylation occurs when a methyl group is transferred to the DNA via a family of DNA methyltransferases. The majority of DNA methylation occurs oncytosines, which immediately precedea guanine nucleotide (ie, CpG site). These CpG sites occur frequently throughout the genome and have been linked to both single-nucleotide polymorphisms (SNPs) and epigenetic changes [2].In particular, DNA methylation may lead to different influences on gene activities depending on the surrounding genetic sequence [3]. Because SNPs near the CpG site may alter methylation levels, the statistical interaction between SNPs and CpG sites may explain varying gene expression across individuals. Prior research shows that DNA methylation in the interleukin-4 receptor is associated with asthma, but this association is further explained by the presence or absence of a nearby SNP [4]. A study focusing on obesity found the interaction between CpG sites in an enhancer region interacts with CpG creating SNP sites in an obesity-risk haplotype, which helps explain obesity/Type 2 Diabetes [5].

As part of GAW20, we were provided access to a data set of methylation, SNPs, and triglyceride levels over 2 time periods, along with numerous related covariates. In particular, the study measured triglyceride levels before and after pharmaceutical intervention. Given the well-known relationship between triglycerides and many different cardiometabolic diseases, including cardiovascular disease [6], we chose to look for evidence of methylation at CpG sites that potentially modulate the impact of nearby SNPs on changes in triglyceride levels.

## Methods

### Sample population and variables

The sample consisted of 670 individuals from a pedigree sample provided as part of GAW20 for whom all analyzed variables were available. We considered 6 covariates (age, observation center, smoking status, mass spectrometry DX client [MSDX] International Diabetes Federation [IDF] score, fasting time at baseline, and high-density lipoprotein [HDL] at baseline) the majority of which were significantly associated with baseline triglyceride (TG) in this sample. The primary response variable of interest was TG level at baseline (visit 1 or 2). For variables with up to 2 measurements at baseline (HDL [baseline], TG [baseline]), we used the average value if both measurements were available, or the only available measurement if only one was available.

### Models

The modeling process was done in 2 stages. The first stage model resulted in a single residual TG value for each person, while the second stage resulted in approximately 700,000 models (one for each SNP that passed standard genome-wide association study [GWAS] quality control [QC] criteria: Hardy-Weinberg equilibrium *p* value> 1 × 10^− 6^, minor allele frequency > 1%, SNP missing data rate < 5%).

In the first stage, we used the *lmekin* function from the *coxme* package in R [7] to predict the change in log-transformed TG levels [*y* = ln (*baseline*)]. In cases where 2 separate TG measurements were available for the baseline, we natural-log (ln)-transformed the data before averaging. Baseline ln-transformed TG levels was predicted by the 6 covariates listed earlier and accounted for the familial relationships in the model through the use of the kinship matrix. We then saved the resulting “residual” value (${r}_i={\widehat{y}}_i-{y}_i$) for each of the *i* = 1,…, 670 individuals in our analysis.

The second stage predicted the residuals (*r*_*i*_^′^*s*) from stage 1 based on the number of minor alleles (*SNP*_*j*_ = 0, 1, 2) and methylation scores (*CPG*_*j*_ ∈ [0, 1]) with a separate model for each *SNP*_*j*_, *CPG*_*j*_ pair using the *lm* function in R [7]. In particular, the second stage model for *SNP*_*j*_, *CPG*_*j*_ pair was:

1 $$ {r}_j={\beta}_{S_j}{SNP}_j+{\beta}_{C_j}{CPG}_j+{\beta}_{S_j{C}_j}{SNP}_j{CPG}_j $$

where ${\beta}_{S_j{C}_j}$ is the estimate of interaction effect between *SNP*_*j*_ and *CPG*_*j*_. *SNP*_*j*_, *CPG*_*j*_ pairs were made by assigning each SNP passing QC to its nearest CpG site, resulting in approximately 700,000 pairs, with some CpG sites assigned to multiple SNPs.

### Statistical analysis

Statistical significance of the interaction term in Eq. 1 was assessed using an *F* test, essentially testing whether the statistical interaction provided significantly more evidence of association with changes in TG levels versus a model with only main-effects terms. Versions of Eq. 1 without the interaction term were also run. We started by using a generally accepted, but stricter and conservative, genome-wide significance level of 5 × 10^− 8^. We followed up this analysis by using a more liberal and exploratory significance level of 1 × 10^− 4^ in our genome-wide interaction analysis.

We followed this genome-wide analysis with a candidate gene study focusing on 18 gene regions (containing 423 unique SNP-CpG sites) that have been shown to be associated with TGs in previous genome-wide association studies via searches at http://www.ebi.ac.uk/gwas. Throughout the candidate gene analysis, we used a significance level of 0.05. As part of the candidate gene analysis we also collapsed all the CpG sites within each gene region (50 kb on either side of the gene) by using 5 different methods (mean, minimum, maximum, median, and sum-squares of the CpG values as the CPG value in the model) to evaluate the potential impact of different ways of summarizing methylation evidence for each SNP. For the SNPs that demonstrated a significant interaction for more than one of the collapsing methods used, we then looked at the interactions between all CpG sites within the region and those SNPs.

## Results

### Genome-wide approach

No interaction term *p* values were significant when using the conservative 5 × 10^−8^threshold. However, 58 SNP-CpG pairs showed significant interactions using the more liberal 1 × 10^−4^significance level. Table 1 summarizes 25 loci that include regions of SNPs that are colocalized and within genes (total of 44 interactions). The median *p* value of the interaction term across all sites was 0.504 and a lambda value of 1.02, showing no inflation of test statistics.

**Table 1:** Summary of 25 loci with significant interactions between SNP and CpG site at the 1 × 10^−4^significance level^a^

Chr	Region that includes SNPs (bp)	# of significant interactions	Smallest interaction *p* value (rs#: cg#)	Nearest genes^b^	Prior GWAS evidence^c^
1	118,384,931	1	8.87 × 10^− 5^ (rs10923477: cg04904531)	*SPAG17*	Cardiometabolic (CM) [10]
1	176,244,329	1	9.17 × 10^− 5^ (rs12078421: cg00529480)	*LOC730102, SEC16B*	CM [11]
1	241,422,395	1	7.97 × 10^− 5^ (rs10926962: cg20140940)	*CEP170*	
1	243,623,480–243,632,536	3	5.45 × 10^−6^ (rs12141949: cg23956499)	*KIF26B, LOC105373266*	CM [12]
2	169,660,479	1	6.32 × 10^−5^ (rs1059261: cg09479286)	*DHRS9, LRP2*	CM [13]
2	228,572,906	1	1.52 × 10^− 5^ (rs11680053: cg13774987)	*SPHKAP, LOC105373918*	
3	62,027,949	1	7.47 × 10^−5^ (rs13094307: cg02315619)	*PTPRG*	CM [14]
3	65,613,288–65,616,308	2	6.31 × 10^−6^ (rs1156024: cg21573947)	*MAGI1, ILF2P1*	CM [15]
3	120,418,689–120,430,978	4	3.03 × 10^−5^ (rs6438504: cg13640423)	*B4GALT4, UPK1B, B4GALT4-AS1*	
5	174,537,630–174,544,483	4	2.17 × 10^−5^ (rs4868496: cg16698913)		
6	71,439,742	1	9.10 × 10^−5^ (rs13196394: cg21039196)	*SMAP1, SLC25A6P6, LOC100419975*	CM [16]
6	127,678,516–127,680,041	2	4.94 × 10^−5^ (rs3798853: cg21774964)	*ECHDC1, RNF146, RPL5P18, LOC105377994, LOC107986641, YWHAZP4*	
10	25,274,245	1	8.54 × 10^−5^ (rs2035888: cg05845435)	*PRTFDC1, THNSL1, ENKUR*	CM [17]
12	70,654,838	1	8.80 × 10^−5^ (rs7300641: cg04586418)	*TPH2, TBC1D15*	
12	90,830,205–90,838,946	5	2.59 × 10^−5^ (rs12318079: cg04373948)	*LOC105369901, LOC105369900*	CM [18]
13	93,851,561	1	6.27 × 10^−5^ (rs9561551: cg21762236)	*GPC6, DCT*	CM [10]
14	31,886,488–32,181,048	5	5.79 × 10^−6^ (rs10141122: cg01642415)	*AKAP6*	CM [11]
14	38,728,932	1	6.17 × 10^− 5^ (rs10140832: cg09400985)	*MIA2, YTHDF2P1, LOC100313942, CTAGE5*	
14	63,424,087	1	3.30 × 10^−5^ (rs4902250: cg04285935)	*SYNE2*	CM [19]
15	22,717,850	1	3.60 × 10^−5^ (rs17785279: cg02476674)	*SNRPN, RPL5P1*	CM [10]
15	74,146,110	1	2.30 × 10^− 5^ (rs7183492: cg19385388)	*TMEM266, NRG4*	CM [20]
16	86,596,651	1	7.26 × 10^− 5^(rs7500034: cg04279689)	*BANP*	
18	25,713,049–25,713,439	2	4.05 × 10^−5^ (rs4799651: cg11963233)		
21	29,961,249	1	5.02 × 10^−5^ (rs2268206: cg06212876)	*GRIK1, GRIK1-AS2*	CM [21]
22	35,532,777	1	7.29 × 10^−6^ (rs736720: cg11855664)	*PVALB, IFT27, LOC105373021, LOC107958578*	CM [21]

^a^Because of linkage disequilibrium structure in these regions each loci can be assumed to have a single independent association (detailed results not shown)

^b^Based ongene_infofile provided by GAW20, supplemented by additional information from dbSNP (http://ncbi.nlm.nih.gov/snp). The nearest genes were always within 50 kb of the most significant SNP

^c^Based on search at http://www.ebi.ac.uk/gwas

### Candidate gene approach

In our data, there are 18 genes (containing 423 SNPs for which data was available) previously shown to be associated with TG levels. Table 2 summarizes the results of fitting Eq. 1 with an interaction term, as well as a version of Eq. 1 without the interaction term.

**Table 2:** Summary of 18 genes with previous evidence of association with triglyceride levels

Gene	Chr	# of significant interactions^a^ (total)	Smallest interaction *p* value	SNP *p*value^b^	SNP location (bp)	Interaction (rs#:cg#)^c^
*APOA1*	11	1 (16)	0.0316	0.296	116,707,207	rs563838:cg24984312
*APOA5*	11	1 (12)	0.0316	0.296	116,707,207	rs563838:cg24984312
*APOB*	2	0 (13)	0.0511	0.124	21,318,003	rs312042:cg23349726
*APOC3*	11	1 (17)	0.0316	0.296	116,707,207	rs563838:cg24984312
*BUD13*	11	0 (13)	0.0866	0.904	116,570,686	rs1784042:cg19442415
*CETP*	16	1 (19)	0.0184	0.502	56,971,665	rs17241126:cg05062620
*CLIP2*	7	0 (2)	0.656	0.662	73,771,865	rs2718277:cg07814763
*DOCK7*	1	3 (75)	0.0261	0.341	63,034,240	rs12122434:cg00161770
*FADS1*	11	1 (16)	0.00430	0.740	61,581,397	rs444803:cg11606466
*FADS2*	11	1 (26)	0.00430	0.740	61,581,397	rs444803:cg11606466
*FADS3*	11	1 (20)	0.00351	0.830	61,710,585	rs1675102:cg16084190
*GALNT2*	1	2 (123)	0.0409	0.605	230,224,139	rs11588595:cg11424376
*GCKR*	2	1 (7)	0.0435	0.0846	27,730,170	rs17706100:cg22903471
*LPL*	8	1 (24)	0.0463	0.497	19,794,163	rs17091651:cg04035597
*MLXIPL*	7	0 (11)	0.106	0.373	73,083,725	rs884843:cg12958963
*OTOR*	20	1 (46)	0.0290	0.469	16,748,375	rs1883698:cg07500957
*PLTP*	20	2 (35)	0.0251	0.983	44,576,565	rs3795126:cg17262492
*TRIB1*	8	0 (8)	0.135	0.638	126,445,881	rs13255114:cg22644321

^a^With a significance level of 0.05

^b^From a model with only main effects terms for CpG and SNP (ie, Eq. 1 without the interaction term)

^c^Duplicates are a result of the overlapping nature of several of the genes

Thirteen of the 18 candidate genes show at least modest (*p* < 0.05) evidence of statistical interaction between nearby methylation values and SNPs within the gene. The most significant SNP is in *FADS3* (rs1675102) and has a minor allele frequency of 0.28. The interaction is such that additional copies of the minor allele lead to a decreased impact of methylation on changes in TG levels.

Table 3 shows the results of collapsing all the CpG sites within each gene region through the minimum method, which uses the minimum CpG value of all CpG sites within 50 kb of the gene. Compared to the other 4 methods, the minimum method resulted in more significant interactions (44) than did the other 4 collapsing methods, which on average only have 23 significant interactions (detailed results not shown).

**Table 3:** Summary of CpG results after collapsing using the minimum method

Gene	Chr	# of significant interactions^a^(Total)	Significant SNPs with > 1 methods^b^	Smallest interaction *p* value (rs#)	SNP*p* value^c^	SNP location (bp)	# of CpGs within region collapsed
*APOA1*	11	0 (45)	1	0.292 (rs633389)	0.381	116,667,337	57
*APOA5*	11	4 (43)	0	0.02564 (rs10488699)	0.00487	116,632,500	80
*APOB*	2	3 (43)	3	0.00286 (rs693)	0.0851	21,232,195	31
*APOC3*	11	0 (46)	0	0.111 (rs632153)	0.119	116,710,239	68
*BUD13*	11	5 (46)	2	0.0252 (rs12279373)	0.0155	116,600,400	63
*CETP*	16	4 (43)	0	0.0199 (rs247609)	0.984	56,973,461	47
*CLIP2*	7	1 (16)	1	0.0143 (rs4298392)	0.791	73,862,441	74
*DOCK7*	1	9 (38)	0	0.0215 (73862441)	0.306	63,049,819	39
*FADS1*	11	4 (18)	1	0.000440 (rs174534)	0.217	61,549,458	107
*FADS2*	11	3 (19)	1	0.00314 (rs174534)	0.214	61,549,458	109
*FADS3*	11	0 (21)	1	0.102 (rs7927548)	0.461	61,690,901	45
*GALNT2*	1	7 (138)	3	0.00154 (rs10779837)	0.194	230,327,568	96
*GCKR*	2	1 (9)	0	0.0480 (rs4665383)	0.0490	27,791,555	32
*LPL*	8	0 (53)	0	0.157 (rs10102876)	0.869	19,779,785	17
*MLXIPL*	7	0 (10)	1	0.0826 (rs7782054)	0.135	73,028,759	98
*OTOR*	20	0 (46)	3	0.0510 (rs16998203)	0.792	16,739,519	20
*PLTP*	20	3 (24)	0	0.0327 (rs11086984)	0.955	44,511,627	91
*TRIB1*	8	0 (32)	0	0.0802 (rs17663005)	0.798	126,464,388	38

^a^With a significance level of 0.05

^b^The SNP was found to be significant with more than 1CpG collapsing method. Refer to methods section

^c^From a model with only main effects terms for CpG and SNP (ie, Model 1 without the interaction term)

We identified 176 unique SNPs in significant interactions for more than 1 of the 5 different CpG collapsing methods as found in Table 4. In total, there are 176 unique significant SNP × CpG interactions. *GALNT2* had the largest number of significant results with 69 interactions, where 1 of the 69 interactions is the most significant with a *p* value of 0.000142. The SNP in this interaction (rs6677241) has a minor allele frequency of 0.026. The interaction results in an increased impact of methylation on TG levels for every additional allele.

**Table 4:** Summary of 176^a^ interaction pairs

Gene	Chr	# of significant interactions^b^(total)	Smallest interaction *p* value	SNP *p*value^c^	CpG *p*value^c^	SNP location (bp)	Interaction (rs#:cg#)
*APOA1*	11	11 (57)	0.00678	0.591	0.310	116,759,824	rs12294191:cg07700644
*APOB*	2	9 (31)	0.00316	0.414	0.518	21,205,457	rs10172650:cg26118553
*BUD13*	11	31 (126)	0.00103	0.787	0.837	116,652,301	rs4417316:cg14371153
*CLIP2*	7	7 (74)	0.00318	0.214	0.983	73,671,288	rs3735504:cg08495433
*FADS1*	11	4 (52)	0.0193	0.0921	0.0831	61,549,458	rs174534:cg07689907
*FADS2*	11	10 (53)	0.00228	0.217	0.432	61,549,458	rs174534:cg11880646
*FADS3*	11	9 (45)	0.0183	0.861	0.690	61,698,488	rs7928792:cg03046346
*GALNT2*	1	69 (288)	0.000142	0.780	0.998	230,337,887	rs6677241:cg03961853
*MLXIPL*	7	16 (98)	0.00220	0.613	0.298	73,041,886	rs6460045:cg03842980
*OTOR*	20	10 (60)	0.00934	0.196	0.581	16,702,501	rs761228:cg07364906

^a^As a result of overlap of gene regions for *FADS1* and *FADS2*, 3 significant interactions are counted twice

^b^With a significance level of 0.05

^c^From models with only the main effect term for CpG or SNP. Refer to methods

## Discussion

Although no significant SNP–CPG interactions were identified when using strict, genome-wide significance thresholds (5 × 10^− 8^), use of a more exploratory approach identified many genes previously shown to be associated with cardiometabolic traits (1 × 10^− 4^). A candidate gene approach, using a significance level of 0.05, identified loci in 13 genes with modest evidence for SNP-CpG interactions on baseline TG levels. Furthermore, by using the collapsing methods, we were able to identify potentially interesting SNPs for additional exploration. Using only these SNPs, our examination of all CpG sites within each gene region resulted in 176 significant unique SNP-CpG pairs. In every case, the SNP-CpG*p* value was smaller than both the SNP and CpG*p* values from the noninteraction model. This suggests that using SNP-CpG pairs may identify SNPs that would not be identified by traditional GWAS techniques. The gene *GALNT2*, had the most significant interactions with 69. SNPs in *GALNT2* were previously identified as associated with TG levels, high- and low-density lipoprotein cholesterol [8]. One study shows that promoter methylation of *GALNT2* is associated with a higher risk of coronary heart disease [9].

There are some limitations to our analysis. First, to manage computational resources, we began by predicting baseline TG levels by kinship and covariates, yielding residuals for each individual, which we used for assessing impact of methylation and genetic variation. Other alternatives to this methodology may exist. We used an exploratory significance threshold for the genome-wide analysis, relative to the vast majority of GWAS-type analyses published today. Although this can lead to more false-positive results, we did find a number of “subthreshold” loci of potential interest suggesting the need for studies with larger sample sizes and more sensitive statistical methods to draw out these loci of interest. The minimum method of summarizing methylation in a region nearby to a gene showed promise, although further work is needed to more fully evaluate the many options. Regardless, leveraging prior biological evidence (eg, via the candidate gene approach) may be of potential effect when testing for SNP–CPG interactions.

## Conclusions

Even with “subthreshold” significance, our results go a long way toward showing the need for statistical models that leverage prior biological information. Our study shows that a mediated effect of SNPs on methylation is a possible explanation for changes in TG levels. With this knowledge, more studies with greater sample sizes can be performed as well as wet lab experimentation to confirm the relationship. As we learn more about the effect an individual’s genotype has on their health, there is greater opportunity for personalized medicine to be an effective treatment strategy.
